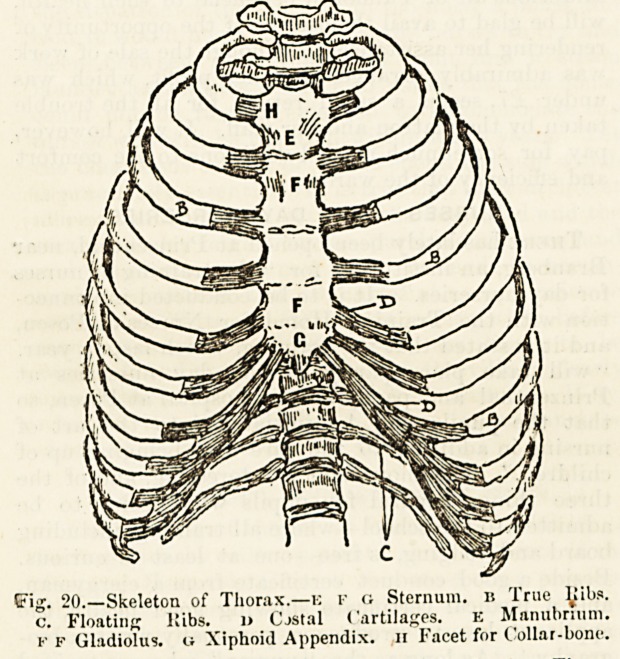# The Hospital. Nursing Section

**Published:** 1902-01-18

**Authors:** 


### The Hospital.

### Nursing Section.

Contributions for this Section of "THE HOSPITAL" should be addressed to the EDITOR, "THE HOSPITAL" NURSING SECTION, 28 & 29 Southampton Street, Strand, London, W.C.

No. 799.—VOL. XXXI. *SATURDAY, JANUARY* 18, 1902.

### Notes on News from the Nursing World.

### THE SCOTTISH BRANCH OF QUEEN VICTORIA'S ENDOWMENT FUND.

IN connection with the anniversary of Queen Victoria's death on Tuesday next, the Duchess of Buccleuch and Queensberry is issuing a special appeal in Scotland on behalf of the Scottish branch of the Jubilee Institute for Nurses. A better way of marking a day which will never be forgotten by the people over whom Queen Victoria ruled, could not possibly have been chosen, and we do not doubt that the appeal will meet with a liberal response on the other side of the Border. The Duchess wisely states the exact position of the case. Of the income of £2,000 a year derived from the Women's Jubilee Offering of 1887, the amount received by the council of the Scottish branch is £400. The amount of the annual expenditure of the Scottish Council is about £3,000, and up to the present time only £2,400 has been raised towards it, leaving a deficit of £600 to be met each year out of a small amount of capital at the disposal of the council. The Duchess asks that this deficit shall be wiped out, and observes that if contributions to the amount of £30,000 could be realised "all anxiety for the future would be at an end." As to the work done under the auspices of the Scottish Council we have already given particulars. It is in order to maintain and extend the work that the appeal is put forward, and we entirely agree with her Grace that "there can be no greater tribute to the memory of our late beloved Queen than the endeavour to further the splendid nursing scheme founded by herself for her poor suffering subjects."

### THE NURSES' CO-OPERATION.

THE annual election of representatives and committee members of the Nurses' Co-operation is now due, and in view of the antipathetic attitude of the committee to the demands of the nurses, it is of obvious importance that new blood should be introduced. All the members and nurses who share this opinion will, of course, make a point of giving effect to their convictions. We believe that the nurses have submitted a list of names of representatives whom they could trust to protect their interests.

### THE WAR NURSES.

THE *Rance* arrived at Southampton on the 8th inst. from South Africa with the following Nursing Sisters on board:—J. Paget, A.N.S.R., and N. G. A. Warner, A.N.S.; both require one month's leave and return to South Africa. The *Orotava* arrived at Southampton from South Africa on the 11th, with Nursing Sister N. Stevens, A.N.S.R., on board; no leave was desired. Superintendent Sister M. Thomas was invalided home.

### NURSES AND VACCINATION.

IN view of the increasing number of small-pox cases in the Metropolis, the Holborn Board of Guardians have decided that no unvaccinated officers shall be employed by them. The reason of the resolution—which it is strange was not put into operation at the commencement of the epidemic—was that one of the nurses at the Highgate Infirmary had refused to be vaccinated. She was, however, foolishly allowed to continue in her position after her refusal, with the result that she contracted the disease, with, of course, the serious risk of communicating it to others. The statistics which were published by the Metropolitan Asylums Board on Monday show that during the past year a very large number of the new staff have joined the small-pox ships and the Gore Farm Hospital, but that not one case of small-pox has occurred. Not a single member of the staff of the hospital ships has ever died of the disease, and not one has even suffered from it for eight years. In reference to the last serious outbreak in 1870-1-2, the Committee appointed afterwards to inquire into the matter, found that amongst the 300 nurses and servants attached to the hospitals which received the cases, there was not a single revaccinated victim of the disease. The only cases were those of nurses whose revaccination in the pressure of the epidemic was overlooked, and who speedily took the disease, and one of a nurse who, having had small-pox previously, was not revaccinated, and contracted the disease a second time.

### SEVEN SUPERINTENDENTS IN TWO YEARS.

IT will be remembered that in our issue of December 21st, we alluded to the extraordinary number of changes in the nursing staff of Shrewsbury Workhouse Infirmary. Since the meeting of the guardians at which this statement was made was held, the two nurses selected to fill the vacancies caused by the retirement of the third and fourth assistant nurses have withdrawn their applications. It is not the least extraordinary feature of the position that the seven superintendents should have been permitted to give up their appointments, one after another, without the guardians taking the trouble to institute an inquiry. The guardians appear to be more solicitous about the interests of the master and matron to whom, we understand, they gave the chaplain's residence, and we hear that the last three or four of their porters have been exalted to the dignity of master of smaller workhouses, in which capacity, we suppose, they will order trained nurses about.

### ST. GEORGE'S HOSPITAL, BOMBAY.

OWING to apathy on the part of the European community in Bombay, St. George's Hospital is still insufficiently staffed with nurses. In October last year it was decided by the managers that the patients in the wards could only be efficiently nursed with

36 nurses, including probationers and five sisters of All Saints'. Yet at the present time there are only 30 nurses and the five sisters available. Not only are more subscriptions required in order to bring the nursing staff up to its necessary level, but there is also a debt of 5,000 rupees on the new quarters of the private nursing staff to be cleared off. It is clear that if Bombay knows its duty to St. George's Hospital it refrains from doing it.

### PARSIMONY AT WALSALL.

The Walsall Board of Guardians seem determined not to provide sufficient accommodation for the nurses. They first rejected a scheme of their own Building Committee, who recommended that a home large enough for 20 nurses should be erected at a cost of £2,400. They next instructed their architect to report upon the accommodation which could be afforded by the acquisition of a villa between the workhouse and the new board-room. But in reference to this, Dr. Fox, the medical officer, reports that the situation of the house is in too noisy a place, and that it would be unfair to ask the nurses to try and sleep between a busy road in front and a steam laundry at the back. He also states that the house could not be further enlarged to accommodate the nurses who will be required within a few years. Yet the Guardians cannot decide to spend the sum which is practically essential. It is true that their architect affirms that the house between the busy road and the steam laundry might, by the purchase of more land and the erection of a new wing, be made to accommodate a matron and 10 nurses at an approximate cost of £800 to £1,000. But seeing that before many years are over 20 nurses are likely to be required, and that the position of the villa is clearly unsuitable, their parsimony is a mistake. Why, when it is admitted that a nurses' home must be obtained, make two bites at the cherry?

### A PLEASANT INNOVATION AT CLUN.

The evening of the 7th of January is not likely to be forgotten by either patients or ex-patients of the Cottage Hospital, Clun. For some time previous preparations had been going on to give them a thoroughly good time at the commencement of the New Year. The rooms and wards had been charmingly decorated by a few friends of Miss Winney, the matron, who had planned the line of operations in a way quite unknown to the town before. The hall was beautified with festoons of evergreens, from which, in careless fashion, the motto "A Happy Year" appeared; a shield inscribed 1902, occupying a central position. The dining-room was adorned with monograms, surrounded by circles of holly, of the medical men connected with the institution. The convalescent ward was also suitably decorated. In the committee room was a fine Christmas Tree, aglow with fairy lights, its branches drooping under the weight of such delights as Santa alone dreams of. Here again kindly thoughtfulness for the patients was evidenced, for while the "little ones" were provided with knicknacks of a most varied character, their elders received more substantial and useful présents. After tea, a concert was given in the convalescent ward; and a farce, which created great amusement, concluded this part of the programme. The tree having been stripped of its presents, the ex-patients departed, giving hearty cheers for the matron; and dancing by the workers concluded the entertainment.

### A CRUEL SUPERINTENDENT.

At Richmond, Yorkshire, on Tuesday, Miss Boultbie, superintendent of the Richmond Nurses' Home, was convicted of ill-treating and assaulting Jeannette Hollins, a girl of 17, whom she had obtained from an orphanage at Bristol, and employed as a general servant at the home. The evidence as to cruelty was conclusive, and the prisoner being convicted, she was sentenced to three months' imprisonment in the second division.

### HOW TO DO IT.

The scheme of the Farnham Board of Guardians for the re-arrangement of the nursing staff of the Workhouse Infirmary having been submitted to the Local Government Board, the latter, in acknowledging the communication, expressed their satisfaction at the careful consideration which the matter had received, and requested that when any further appointments of nurses or probationers are made, they may be reported for the Board to sanction. We quite concur in the opinion of the guardians, that the letter "inferentially" shows that the Local Government Board approve the report sent in. If they had not done so, they would certainly have pointed out defects. We believe that if only Boards of Guardians would adopt a reasonable policy, and do their utmost to level up the nursing arrangements in the workhouse infirmaries to present-day needs, they would find the Local Government Board easy to satisfy.

### PRIZES FOR ESSAYS.

The members of the Executive Committee of the Dublin Nurses' Club offered three prizes for the best essay on "Enteric Fever." The first was won by Miss Daisy Young, of the City of Dublin Nursing Institution; the second by Miss Naile, and the third by Miss Cunningham—both of Sir Patrick Dun's Hospital. Miss Young's paper was read at the last meeting of the club, and an interesting discussion followed, in which many of the members joined. It was announced that two more prizes were offered for papers on different subjects, and it is hoped that they will also prove of much benefit and instruction to the members.

### PROGRESS AT BARNSTAPLE.

The Barnstaple Board of Guardians were not allowed to adopt a very necessary proposal by the visiting committee without an attempt to maintain an arrangement which cannot be defended on any grounds whatever. It has been the custom for the male patients in the infirmary to act as nurses, the only trained nurse employed by the Guardians having as much as she can do to look after the female sick. This custom is, of course, as bad as it is hoary, and the majority of the Barnstaple Guardians have come to the decision not to continue it. The plan for allowing the 22 male patients to be looked after by old inmates was justified on the ground of economy, but this was disposed of by the rejoinder that though the Guardians are anxious to avoid unnecessary expenditure, they are also desirous of properly nursing the people in their care. Perhaps the citation of the fact that the Local Government Board forbid patients to be employed as nurses also carried weight.

At all events, there is, at last, to be a nurse for the male sick at the Barnstaple Workhouse Infirmary.

### A NURSING INSTITUTE AT RYDE.

THERE has just been opened a new nursing institute in the Isle of Wight, at the instance of Mr. C. H. Wheatley, as a memorial of his little child whom he lost a few years ago. The building has hitherto been known as St. Augustine's, and is a large and well-appointed house in close proximity to Ryde. Here six medical, surgical, or convalescent cases will be received and looked aftor by a sister, under whom is a staff of fully-trained hospital nurses. We observe that a local paper states that until now a Ryde medical man has had to send to Ventnor or Southampton when he required the services of a trained nurse. The institute should therefore be of service to the fairly-well-to-do residents of the neighbourhood. But the poor of Ryde have still apparently no chance of good nursing when sickness overtakes them. Is there no one ready to follow the good example of Mr. Wheatley, and arrange that Ryde shall have at least its district nurse for those who are not likely to benefit by the new institute ?

### AN ENTERTAINMENT BY DISTRICT NURSES.

IT is always pleasant to hear of district nurses giving their time and trouble for the benefit of the poor under their charge even after the hours of nursing are over. An entertainment has just been organised by two nurses at Bristol, who must have felt cheered and gladdened by the success of their undertaking, and the gratitude of their guests. The money had been collected quite independently from friends, and on Friday evening, January 3rd, 280 children, living in one of the poorest parts of Bristol, were invited by Nurses Ocker and Bowman—a past and present district nurse of St. Matthias and St. Clement's—to a Christmas-tree entertainment held in the St. Matthias schoolroom, lent for the occasion by the vicar. The tree was prettily lighted with coloured candles, and whilst they were burning the children stood round and sang carols. Then the toys were distributed, and fun and laughter and noise became striking features of the entertainment, showing the pleasure of the little ones. Before they left, each child received a bun, an orange, and a cracker.

### ROYAL NATIONAL PENSION FUND FOR NURSES

THE Secretary of the Royal National Pension Fund for Nurses desires to acknowledge with thanks a donation of £1 from "A Hospital Sister." We need hardly add that contributions of this kind, whether sent anonymously or otherwise, are always acceptable at 28 Finsbury Pavement.

### DEARTH OF NURSES AT BRISTOL.

WE have not the slightest doubt that the Hospital Committee of the Bristol Board of Guardians have rightly arrived at the conclusion that the short annual holiday given to their nurses is a primary cause of their difficulty in filling vacancies. There can be no question about the existence of the difficulty. At present there are vacancies for three charge nurses and two assistant nurses at the workhouse hospital, and the committee were so hard pressed in obtaining nurses during last year, that they found it necessary to promote six probationers to fill the position of assistant nurses before they had completed their three years' training, and four assistant nurses to fill the position of charge nurses. As to the latter there is nothing to be said, but the excellent principle of promotion may be pushed too far, and the opinion of the committee that the early promotions of too many probationers is not desirable, is quite sound. So also is the view that a fortnight's holiday is too short ; and although a trade-union member of the Board of Guardians thinks otherwise, we hope that, in their own interest, his colleagues will adopt the suggestion of a lady member, and consider the advisability of increasing the length of time.

### FALMOUTH HOSPITAL.

FOLLOWING the sale of work which took place last month in aid of the Falmouth Hospital and Dispensary, the matron is anxious to get up a small bazaar to be held during the summer in the grounds of the hospital. It is possible that there may be among our readers persons who, having found the salubrious air of Falmouth beneficial to their health, will be glad to avail themselves of the opportunity of rendering her assistance. Although the sale of work was admirably arranged, the net profit, which was under £7, seems a small return for all the trouble taken by the matron and her staff. It will, however, pay for some much needed additions to the comfort and efficiency of the wards.

### "NURSES" FOR DAY NURSERIES.

THERE has lately been opened at Prinzenthal, near Branberg, an institution for "the training of nurses for day nurseries." It is to be conducted in connection with the Training Home for Nurses at Posen, and it is stated that the training, which lasts a year, "will take place partly at the day nurseries at Prinzenthal and partly at the hospital at Posen, so that the pupils will be initiated into the art of nursing in addition to the care and bringing up of children." The movement is interesting, and of the three things needful for pupils who desire to be admitted to the school—where all training, including board and lodging, is free—one at least is curious. Beside a good conduct certificate from a clergyman, and a medical certificate showing good health, the applicant has to produce a personally written biography ! As long as the "nurses" who are trained at Prinzenthal do not afterward pose as "trained hospital nurses" on the strength of the instruction they receive in the hospital at Posen, the new departure can only produce excellent results.

### SHORT ITEMS.

THE second sessional lecture under the auspices of the Royal British Nurses' Association will be delivered by Sir Thomas Lauder Brunton at 10 Orchard Street, W., on Tuesday next, at 5.30 P.M. Subject : "Spiritualism and Haullucinations."—At the usual fortnightly meeting of the Metropolitan Asylums Board on Saturday last letters were read from the Local Government Board sanctioning the appointments of Miss S. A. Villiers and Miss A. Thomas as matrons of the Fountain and Park Hospitals respectively.—The patients of Stapleton Union Infirmary, Bristol, passed an enjoyable evening on January 3rd. Thanks to the kindness of the nurses, they were provided with an excellent tea, which was followed by tableaux, songs and recitations, also by the nurses, the medical officer presiding.

### Lectures to Nurses on Anatomy.

By W. Johnson Smith, F.R.C.S., Principal Medical Officer, Seamen's Hospital, Greenwich.

### LECTURE IX.—THE THORAX.

The skeleton of the thoracic cavity or chest (fig. 20) which encloses the lungs and the heart is a large conical case compressed from before backwards in the middle and bulging on either side and below. This osseous cage is formed behind by the 12 dorsal or thoracic vertebræ, in front by the breast-bone or *sternum*, and by 12 ribs on each side. The cavity communicates freely with the deep structures of the neck by its small upper aperture, and at its large lower aperture or base is shut off from the cavity of the abdomen by a thin and expanded muscle termed the diaphragm. The long and narrow intervals between the ribs—*the intercostal spaces*—are closed by two thin layers of muscle—*the external and internal intercostal muscles.*

The *ribs* increase in length from the first to the seventh and then rapidly decrease to the last of the series. Each rib is attached behind to the spine, but the anterior end of each bone does not reach the breast-bone. The large gap thus formed on either side is bridged over, completely in the upper seven ribs, incompletely in the next three, and not at all below these ribs, by bars of a gristly or cartilaginous

Fig. 20.—Skeleton of Thorax.—E F G Sternum. B True Ribs. C. Floating Ribs. D Costal Cartilages. E Manubrium. F F Gladiolus. G Xiphoid Appendix. H Facet for Collar-bone.

material, softer than bone, and pliant and elastic. These bars are called the *costal cartilages* (fig. 20 D). In each of the upper series, termed the *true ribs*, including the first and seventh and the intervening ribs, the bar of cartilage is attached by one end to the sternum and by the other end to its corresponding rib, so that each continuous bar of rib-bone and rib-cartilage is separate from the others. The cartilages of the next three ribs—the eighth, ninth and tenth are welded together, and also to the cartilage of the seventh rib, and do not reach the breast-bone. The anterior extremities of the eleventh and twelfth ribs are tipped with cartilage, but are quite free and disconnected both from breast-bone and the other ribs. For this reason the two last bones are called *floating* ribs.

On examining a typical rib—the sixth or seventh—we find the posterior extremity slightly expanded to form the *head* of the bone, the free surface of which in a recent state is covered by cartilage. This cartilaginous surface, it should be observed, is separated by a cross ridge into two articular facets. The end of the rib, as we found when discussing the spine, articulates as a rule not with the body of a single dorsal vertebra but with two vertebræ. Between the head of the rib and a knob of bone about one inch in front of it, is the *neck*, which, as a neck should be, is rounded. The knob of bone also presents a facet for cartilage which joins a similar facet near the tip of the transverse process of each dorsal vertebra. The rib now suddenly takes a change in direction, and is curved forwards and at the same time downwards. The part of the rib where this double curvature begins is called the *angle*. The bone is no longer cylindrical, as in the neck, but flattened in front and behind. The upper edge is even and rounded; the lower is sharp and thin, and covers a well-marked groove which runs along the rib near its lower margin and is occupied by blood vessels and a nerve. If we bear in mind this difference between the upper and lower edges and also the direction of the curves of the bone, we shall be able to distinguish a right from a left rib. The anterior end of our rib to which the costal cartilage is attached presents an oval and concave surface of rough bone.

In the ribs as in the vertebræ certain bones present marked peculiarities. The first rib is very short, much curved, and flat, the edges being in front and behind, and the broad surfaces above and below. If we can obtain this rib for close examination we should look for a small knob of bone near the inner margin and at the junction of the anterior with the middle third of this margin. This knob will be found the guide to two grooves on the upper surface of the rib marking the course taken by the large blood-vessels—the *subclavian*—which pass from the neck to the arm. The last two ribs are short and stunted and have no necks. The posterior articulating surface presents only one facet—not two—in the first rib and the last three ribs. These ribs, consequently, articulate with a single vertebra and not, like the majority, with two.

A "broken rib" may be the result of direct violence, as, for instance, the kick of a horse, or of indirect violence as in squeezing of the chest. In the former instance the ends of the two fragments are forced inwards, and there is a risk of serious injury to the structures contained in the thoracic cavity. In fracture from indirect violence the broken ends are forced outwards, and this form of injury, even though several ribs be broken, is not in itself so serious as that from direct violence. It is occasionally very difficult to make out whether a rib has been broken or not, but such injury should always be suspected when the injured person complains of a darting or stabbing pain at one part of the chest, when he breathes with difficulty, and when he cries out on attempting to "take a deep breath."

The thorax is completed in front by a long and flat bone called the *sternum* (fig. 20 E F G), which covers and protects the large blood-vessels connected with the heart, and a portion of the heart itself. This is not a single bone, but is made up of pieces varying in number at different stages of life. In the infant there are six distinct bones arranged one above the other. In the "grown-up" subject we find that there are only three pieces, the second to the fifth having been fused together like the five segments of the adult sacrum. In a person of advanced age the number of pieces may be reduced to two, and in some rare instances even these two portions may coalesce and form a continuous bone. The sternum (fig. 20) of the adult presents under usual and normal conditions two distinct pieces: a broad upper piece (E), a longer and narrower middle piece (F F); both these being composed of bone, and a small appendage lying just below the letter G in the diagram, composed, not of bone, but of pliant cartilage. This appendage or process varies much in length; it may be directed backwards or forwards, and is sometimes forked at its lower extremity; but in most persons is short, straight, and single-pointed. The older anatomists influenced by an idea that this sternum resembled a Roman sword gave the name of *manubrium* to the upper piece, of *gladiolus* to the long middle piece, and of ensiform or *xiphoid appendage* to the terminal piece of cartilage. In the sternum as in the sacrum the original separation of the bone into segments is indicated by transverse ridges. The most marked of these, and one that can be readily felt under the skin, runs across the bone between the right and left attachments of the second ribs. Along each lateral margin of the whole bone are seven notches on which are fixed the first seven costal cartilages on either side. In addition to these there is a pair of larger notches at the upper part of the manubrium—one at each of the upper angles, which receive the inner ends of the collar-bones (H).

### Beyond the Seas: Nursing in Cyprus.

BY AN OCCASIONAL CORRESPONDENT.

REMEMBERING the difficulty I had in finding out anything about Cyprus before coming here, it is possible that some readers of THE HOSPITAL may be interested in a glimpse of life in the island as seen by a colonial nurse. The first view is very interesting, for the low line of buildings along the shore, broken by minarets and palm-trees, has a fine background in the central range of hills which reach a height of 5,000 feet at some distance in the interior and are crowned with snow during several months of the year. It was the beginning of the hot weather when I arrived, and the drive from Larnaca, the nearest seaport, to Nikosia, the capital, is at that season, one of the most dreary imaginable. Scarcely a tree of any kind is to be seen; at first hillocks of a sandy hue, varying in size from tiny mounds to small hills, lie on each side as far as the eye can reach. Sometimes they are covered with dry thorn bushes of the same dull tint. Even the villages, one or two in number, which are passed during the four-hours' drive, being built of mud bricks, and consisting of low, flat-roofed houses, scarcely relieve the monotony of the picture, and after the last few miles of dreary plain in the midst of which Nikosia lies, the town looks like an oasis in the desert with its green belt of trees. It is a town with a rampart all round and a moat planted with well-grown shade trees and pines, above which rise the tall date palms and minarets that give it such a picturesque, Eastern look. Inside the town everything is a strange mixture of East and West—narrow streets with overhanging balconies nearly touching one another, strings of loaded camels, bullock carts, flocks of goats with bells tinkling at their necks, being driven round to, and milked at, the customers' doors. In the bazaar, tailors, carpenters, shoemakers, sitting in their open-fronted shops, busily ply their respective trades. Turks in their long flowing garments, and women with veiled faces mingle with the up-to-date Cypriots in English suits and Paris frocks. Bicyclists are also present, and ordinary English dog-carts and carriages have often to make way for the four-horse diligences (the horses harnessed abreast), in which long journeys to the distant towns are performed. Nearly all the poor villagers ride on mules or donkeys, sitting as far back on the animal as possible so that at each movement it looks as though they must fall backwards or slip down over its tail.

### LAZARUS FLOWERS.

The hospital is outside the town, where the trees have straggled along far past the moat and hedge the roads on either side, making quite a green suburb, where many English officials have their houses, principally built in bungalow style and surrounded by pretty gardens. There is an avenue of the graceful pepper-tree from the hospital to the gate, and the whole of the enclosure, though dusty in hue for six months in the year, is a blaze of yellow marguerites in the spring. The people call the marguerites "Lazarus' flowers" for Lazarus is supposed to have gone to Cyprus after his resurrection, and to have lived there until his death. His tomb is shown in a vault under the old church of St. Lazarus in Larnaca—and really the appearance of this lovely yellow carpet of flowers, so soon after the rains, quite accounts for their popular name. Our little church, too, is surrounded by quite a flower-garden at that time, and makes the prettiest picture imaginable.

### THE WARDS.

The hospital is a stone building of three blocks, one administrative, containing offices, operating room, rooms for private patients, etc. The English matron and private nurse also have their rooms here. This is a two-storey building—as is also the men's block—which is connected with it by a corridor, and consists of two large and two small wards. The former contain 11 beds each. The upper floor is kept for the Zaphteihs, or military police, two of whom act as orderlies in the men's wards. A continuation of the corridor brings us to the women's and the eye block, a one-storey building with several small wards and rooms for the native nurses, of whom there are four. Two of these have been here for some years, but the other two are probationers. One is being trained for a small district hospital, to which she will shortly be passed on.

### OUR PATIENTS.

The patients are principally Greeks and Turks. Every town and village has its Turkish and Greek quarters, its Greek churches and Turkish mosques. There is also a Jewish community some miles outside Nikosia, and patients coming from there can sometimes only speak Hebrew or a little French. We also get Armenians, so that the variety of languages in the wards is great. Cyprist Greek, however, is spoken by nearly all our patients, so we have to pick up a certain amount of it as quickly as possible. Some of the nurses and orderlies speak a little English, and the other officials speak it well. The doctor, of course, is English. There is a large out-patient department and dispensary, which is well attended by both Turks and Greeks. The Turkish women are very picturesque with their white yashmaks, which cover them from head to foot and are held across the face so that only the eyes are visible. Some yashmaks are bright colours—red, yellow, mauve, or green—and even striped and checked, but the white are the prettiest. The patients sit and lie around under the trees when they overflow the benches; some even bring their beds to lie upon, which more nearly resemble wadded quilts than beds. Those who have come from distant villages tie up their donkeys beside them. The donkey's saddle is usually a bright-coloured blanket, folded, and the women ride astride like the men. Greek village women generally wear rather short skirts and high leather boots, nearly to the knee, into which their full trousers are tucked, so that the style of riding is quite convenient.

### BABIES AND OPERATIONS.

Many babies are brought as out-patients—bad eyes being common. The poor little things are wound up like mummies, with their tiny faces only visible. They all wear caps, and are so dirty, soap and warm water is the general prescription in addition to any other treatment. The eyes are usually carefully blacked all round with a mixture of charcoal and oil by the mothers. Many operations are done at the hospital, nearly all of which are very successful. Wounds heal most readily out here, which is fortunate, as the natives are rather excitable, and cases of stabbing and cutting are frequent. At one time we had five cases in the ward at once, with police in charge night and day, as the patients had all to go before the court for trial as soon as they were convalescent. Patients who die and are taken out to be buried are dressed in their every-day clothes, including boots, hats, or bonnets, and are carried on an open bier to the grave, followed by a crowd of mourners weeping and lamenting in a kind of sing-song at the top of their voices. Even before they die the friends will often sit on the ground outside the hospital, and howl and beat their breasts and tear their hair.

The Climatic Advantages.

It is very hot here from May to September inclusive, but the climate is dry and healthy. Along the coast it is a moister heat and more trying. The English regiment stationed at Limassol goes to the hills for the summer, as do the Governor and many of the English residents, where they mostly live in wooden huts or under canvas. It is a most exhilarating air on Mount Tröodos, where they camp, but the change from a high temperature in the day to a very low one at night is dangerous, unless one is very careful with regard to clothing. The soldiers have orders to change from khaki to cloth suits at sundown. The heat is quite bearable on the plains, however, even during the hot months, and the winters are very pleasant. About three months of the year it is really cold, and we are glad of fires and warm clothes; but there are very few dull days, and, taking it as a whole, wind and dust are the most trying features of the Cyprus climate, whilst the mosquito and sandfly are most persevering in their attentions during the summer months and quite prevent anyone feeling dull or lonely.

### Sick-Room Cookery.

By Maud Mason.

### Dainty Modes of Cooking Eggs.

Eggs, like milk, are often spoken of as a "complete" food —that is, they contain all the elements of the blood and provide all that is necessary for the development of the young chick—but in the latter case it must be borne in mind that some of the essential materials for growth are dissolved from the shell during the process of incubation. As a separate food for human beings they are deficient in carbon. They do, however, provide a concentrated and nutritious food, being rich in nitrogenous matter, and in sodium, iron, and phosphoric acid. The white of egg consists mainly of pure albumen, with a small amount of fat and salts, while the yolk contains a large quantity of fat as well as albumen and salts, and therefore has a greater feeding value. Eggs offer an easily-digested food if taken raw or lightly boiled, but if boiled hard they are exceedingly difficult of digestion. According to Dr. Beaumont's experiments, raw eggs were digested in two hours, soft boiled in three hours, and hard boiled in five hours. The ordinary cook would be very much at a loss for everyday cookery without the use of eggs, and they may be extensively used in invalid cookery, though of course in some diseases they are entirely forbidden. It is a *sine quâ non* that where eggs are given and allowed they must be given in the most easily digested form possible. The lightest way in which they can be taken is to beat up the yolk of egg with a little sugar and hot water, this is called "lait de poule." Another method may be adopted where it can be tolerated: beat up the yolks of two eggs with a teaspoonful of sugar, add two teaspoonfuls brandy, sherry, or port wine, whip up the whites stiffly, add them to the yolks and stir well together. When the white of egg is to be beaten up by itself, it is more readily done by adding just a small pinch of salt, as for instance in the following cordial: Beat up the white of egg to a stiff froth, add a few spoonfuls of cream and a little brandy. When milk may be taken along with the egg, the egg should be beaten up, the milk boiled, and then allowed to cool a little, poured on to the egg, sugar and wine added to taste.

### How to Poach.

Now, with regard to the manner in which eggs may be cooked. To poach them is probably the best plan and serve them on a dainty piece of buttered toast. Poached eggs are cooked in water, never in fat, which hardens them. It is not the easiest thing in the world for the novice to poach an egg; either the egg spreads too much or the yolk splits when the egg is cracked or slipped into the water. First a word as to cracking the egg. Give it a sharp hit on the side of a cup, and then hold the shell so that the white runs out and the yolk remains in the shell, and then it can be let down into the cup easily—a cup holds the egg more compactly than a saucer. Then as to the pan in which to cook it; choose a low saucepan, not a frying-pan, there must be a depth of water; add salt to the water, let the water boil, draw the pan to one side of the stove, and carefully pour in the egg, tilting the pan a little, so that the egg keeps together and does not run all over the bottom of the pan. Just let the water bubble for about four minutes, then slip a fish slice under the egg, lift it out, trim off any untidy edges, and place on the toast, which should previously have been prepared. If properly done there should be a slight covering of white over the yolk. The difficulty which I have observed is experienced in managing this properly is my only excuse for having given such minute directions. An egg may be delicately cooked in the shell by placing it in a pan of boiling water, covering with a lid, and then left on the side of the stove, without allowing the water to boil again, for five minutes.

### When the Digestion is Stronger.

A very dainty way of serving eggs when the digestion is stronger is to cook them and serve them in little tiny dishes made for the purpose; they are of fire-proof china, and are like miniature frying-pans in shape, only the sides are straighter, and they just hold an egg. The cost of these little dishes is about 9d. each, but a little patty-pan may be employed if you do not possess one of the china dishes, and here there is no limit to the variety of methods. In all cases the eggs are cooked in the oven or on the stove. A little butter is first melted in the dish, the egg broken into it whole, the flavouring added, and the egg allowed to cook till it just becomes set. They are very delicious cooked plain, and any of the following may be used to form a pleasant change: Chopped green parsley, just a sprinkling of dried herbs, a spoonful of tomato sauce, a mushroom minced and placed under and over the egg, with or without the faintest suspicion of onion, a little minced ham, and so on *ad infinitum*.

### Omelets.

For the cooking of eggs in omelets, of which there is a very large selection, I must refer you to any good cookery book. Take care to steer clear of the accusation often hurled at English cooks—that they produce something which resembles a pancake or a piece of wash-leather—their lightness should be their chief recommendation.

I may mention that it is often a very satisfactory way to serve a poached egg in clear soup. This soup is said to have been invented after one of the Imperial hunts at Fontainbleau, when the Empress, who was very fatigued, declared she could only eat a new laid egg. The chef was equal to the occasion, and served a consommé á l'Impératrice, that is, he added poached eggs to his soup, inventing a new dish, which was the means of dissipating his Royal mistress's fatigue and stimulating her appetite.

### Lectures to Head Sisters.

By E. MARGARET FOX, Matron of Tottenham Hospital, N.

LECTURE III.—(*Continued from page* 153.)—A HEAD SISTER'S DUTIES TOWARDS HER PATIENTS.

WITH regard to your patient's own personal clothing, it will save you a great deal of trouble in the end, as you all know, if from the very first you are careful of it. Empty the pockets in the presence of the patient or his friends, and see that all money or valuables are at once consigned to safe keeping. Allow no large sum to be kept in the lockers. Make an inventory of the clothes if they are to remain in the hospital; examine them to see if they are dirty, and, if so lose no time in getting them baked. Do not leave them lying about the ward, bath-room, or kitchen for an indefinite time, but on the other hand, do not bundle them away without due regard to spectacles or suchlike small unconsidered trifles which, if not at hand, the patient is certain to want when it is most inconvenient to fetch them. See that all his property goes with him when he leaves, for you all know how very annoying it is to have a patient calling at the hospital again and again for something he says he has left behind which nobody can find.

Before sending home by the friends, the clothing worn by an enteric patient, take care that it is baked first, otherwise it may help to spread the disease, and see that the clothes are returned to the hospital in time for the patient to wear when he is convalescent. As soon as the doctor orders him up, tell the friends to bring the clothes. Sometimes, patients who should be walking about the ward in readiness for going home, are kept on the couch in a dressing-gown, or go about insufficiently clad for quite a long time, because the head sister has forgotten to remind the friends about the clothes. This may seem a little thing, but after all it makes greatly for the comfort and well-being of those under your care if you do not consider the details, even of their clothing, beneath your notice. Bear in mind always that your chief aim with regard to your patients from the moment of their admission until their departure is to facilitate their speedy recovery in every way.

To this end you strive to procure for them absolute rest—of mind, by removing all known sources of irritation and annoyance—of body, by well made beds, carefully padded splints, suitable food and clothing, etc. In all your treatment of them, their ultimate good must be your aim, and if you get into the way of always thinking what is best for them—if your mental attitude towards them is always an unselfish one, then your daily services cannot help being acceptable to them, and you will become more and more helpful with each week of expanding knowledge and experience.

There are so many things you do for your patients, as well as the quiet routine of daily work. Think, for instance, of the effect it will have on that young servant girl, to see how deftly and tidily the ward is being swept and dusted, the fires made up, or the meals served. It will be a real object lesson to her on the way to do her work. Or that mother of a large family. She hears your kind, gentle words. She marks your quiet patience under circumstances of great provocation, and mentally registers a vow to imitate your self control. The children's visitors see your kindnes towards the little ones, and their quick responsiveness Who knows how often the memory of it checks the hasty word or blow, because the "sister at the hospital" never does it, and yet the children obey her readily when she speaks.

Those young lads and girls in both men's and women's wards. Do you think they do not watch to see how you behave, day by day, especially at times when they know you are not under any immediate supervision? While the house surgeon is making his rounds they are observing you closely, and are quick to see whether you are acting in a quiet, natural manner, your thoughts on your work and not on the impression you think you are making, or whether you are frivolous, self-conscious, or anxious to air your knowledge. They cannot help judging you, and pretty correctly too, and knowing as we do that the behaviour of these same lads and girls towards each other leaves so much to be desired, it behoves one and all to be living examples to them of a more excellent way of conduct, teaching them self-respect and gentler manners without one actual word being said to them on the subject.

The ward represents to them for the time the nucleus of their daily lives. They mentally compare it with their everyday environment of shop, house of business, factory. Let them see you thoroughly businesslike in all you do, punctual, tidy, clean, quick and methodical. Your manner to each other, to those working under you, to the ward maids and scrubbers, will represent to them what theirs should be to their mates, their subordinates, and those in authority. Do let it be such as may safely be carried away with them, and reproduced, perhaps, unconsciously, into that shop or factory. They will go away, feeling there is no need to give rough words, rude answers, curt commands to each other after two or three weeks spent under the influence of a gracious, courteous woman who has been able to maintain order and discipline quite easily by means of quiet requests spoken in carefully modulated tones. All day long they lie there, and if you are living up to your ideals they will notice that you never seem to be doing anything you are ashamed of and that you never hastily change your occupation because a doctor, your matron, or a visitor enters your ward. They see your quiet reverence at prayers and the absence of anything like chatter or levity in the ward kitchen during the chaplain's visit, your courteous reception of clergymen or ministers of any denomination whatever who call to see them personally. They notice how neat you always are in dress and appearance and that it is possible for a woman to look her very best in the severe simplicity of uniform. Some of the "white light that beats upon a throne" is on you, whether you will or no, and your simplest actions often assume quite undue proportions. If you have ever lain ill on a hospital bed, you will know how you learn to watch the faces of those around you; how sensitive you become to any change of temper or manner in your nurses; how a hasty word stings and rankles long after its speaker has forgotten uttering it, and how quick you are to notice any unwillingness to perform a service for you. I do not think we can overrate the extent of our moral influence over our patients, and if we have their ultimate good always before us, we shall ever be on our guard to be ourselves what we would have them imitate.

(*To be continued.*)

### To Nurses.

**WE invite contributions from any of our readers, and shall be glad to pay for "Notes on News from the Nursing World," or for articles describing nursing experiences, or dealing with any nursing question from an original point of view. The minimum payment for contributions is 5s., but we welcome interesting contributions of a column, or a page, in length. It may be added that notices of appointments, entertainments, presentations, and deaths are not paid for, but that we are always glad to receive them. All rejected manuscripts are returned in due course, and all payments for manuscripts used are made as early as possible after the beginning of each quarter.**

### Night Nursing: a Few Words to "Pros."

By a Fever Nurse.

This may well be called the age of "specialities." A "specialist" must be consulted because he has made some particular disease, or condition, or treatment a "special" study. In the nursing profession the whole is a huge affair and attainment, every one of its many branches to be at least looked into and learnt something of; yet from experience one might almost say that a really good night nurse is "a speciality."

### The Main Object.

But to "specialise" and be practical, how must the night nurse cultivate her "speciality"? Her main object is to secure rest and sleep for her patients; and true nursing, and caring, and helping, and thought, and ingenuity, and giving up of self were never better called out, for it is in the hours of darkness and of suffering wakefulness that trifling afflictions are so hugely magnified—be it only a ruck in a sheet, a pillow slipping away, or a few crumbs in the bed—they take not a second to right, but are sometimes left unremedied from want of willingness on the part of the nurse. "Wait a minute," or, "I will come presently," acts on the nerves of the weakly patient, till the crumbs are pebbles and the rucks rocks.

### "Fads."

To induce sleep there are many small things which the most junior probationer may bear in mind. Learn to remember the "fads" of each patient. One likes light clothing, another heavy, or a double fold of blanket over the feet; a hot-water bottle at the foot of the bed; absolute darkness, or some light; a screen by the bed to hide the vastness of the public ward for some of the aged or nervous; or, on the contrary, the removal of the same should its presence cause annoyance. If sleepless, try some alteration of position—a pillow under the knees, a small one under the back, or so arranged as to take the shoulders cosily, re-make the bed if the patient may step out or be lifted, bathe the head and back of the neck, apply a bandage to the temples drawn tightly round the head, and be sure that sheer hunger is not the cause of the wideawake state. If allowable, take the trouble to make a cup of cocoa, gruel, bovril, or, perhaps better still, bread and milk—not sop. The other patients must not be disturbed through your ministrations to one, though in some cases it is a real advantage to thoroughly rouse one "who cannot get to sleep," and have a quiet friendly general chat. That much maligned refresher—a cup of tea—has more than once induced sleep by its slightly stimulating properties, and in private nursing the fact of quietly preparing such a tiny meal, especially if it be got ready in his room, has a curiously soothing and entertaining effect on a sleepless patient, who very likely will afterwards fall asleep and awake surprised, to find the day well in. These, and fifty other ways of attending to the comfort of one's poor things crowd into one's mind; but it will scarcely be credited how frequently such small help is unafforded, all for want of thought.

### Self-Control.

A night nurse must practise self-control. It does not always seem to be borne in upon her that the wakeful and sickly are her "special" burden. She should, therefore, whilst giving her cheerful presence to each in her care—for there is seldom a hospital ward without some convalescents or such as sleep the night through—refrain from that light and frivolous talk with the hale and hearty, whilst poor little Tommy, with that terrible back, is calling for nurse, or "the fracture" two beds below is waiting his chance of getting his heel-pad readjusted. Nor is it seemly for nurses to keep the "H. P." or "H. S." conversing on every topic of the day, sparring at each other and cracking jokes until patients who had awoke with a temporary want which a moment's service from nurse would satisfy, are thoroughly aroused, not to sleep again till the 3.30 or 4 o'clock clatter of mugs and plates, preparing for the early breakfast, forbids more rest for at least some hours.

### Quiet Deportment.

Nor does every probationer realise at once the importance of quiet deportment on her own part. The ward-kitchen, often so immediately near the ward, is visited by a fellow-nurse. Talk and laughter go on at the highest pitch of the voice, tea-cups are rattled, spoons tossed into saucers, toast scraped with energy, cupboard doors thrown open with a bang, door handles allowed to jerk back, taps to run, coals to fall, even fire-irons to slip and slide—every noise being emphasised in the stillness. Even the very waiting on the patients is done by some as though it were in the middle of the day and the whole world astir. Bed pans are brought in two together, rattling the whole way up the ward, bowls and receivers set down on the locker with a will, scissors and instruments thrown clattering and jangling into enamel vessels, or the heedless nurse makes five journeys to a bed in getting together her necessary dressings, where one, or at the most two, should do, and, on occasion, does not omit to bring the flaring night lamp at the commencement of her prolonged preparations instead of at the last moment when all is ready. And these trifles—so trifling indeed that younger readers may smile at their foolishness—but that soon run up and make a very serious deduction from the efficiency of a night nurse, can be obviated by a little thought. The overlooking of these matters hardly comes nnder the head of training, technically speaking. It is a trait of character, showing want of fellow feeling. Any woman, however, aiming at being a "trained nurse," must see to it that she is not above learning to do to others as she would be done by, and a nurse once warded will soon find out the irritation and annoyance which a careless nurse can cause.

### The Ordinary Work.

As to the ordinary nightly work of a night nurse much could be said, but it is rather the "speciality" of night nursing which I have in mind—the main object—and the "special" one being to secure "a good night" for one's patients. I have in mind the unfortunate aptitude of night nurses for keeping their patients awake, by inattention, laziness, carelessness, self love, and more often by bare want of thought—nothing worse. Of course in respect to the special treatment, as in respect to the giving of ordered drugs and hypodermics, the nurse carries out the directions of the doctor, taking care that sleeping draughts are not given before the patient is made thoroughly comfortable in every way. A good nurse will bear each detail in mind, and ask particulars as to the feeding and medicines of the subject of insomnia; she will not let the doctor retire for the night, or go back to his home at some distance, only to be disturbed a couple of hours later because she has forgotten to ascertain whether he wishes his patient to be awakened for this or that dose. There is a kind of rule quoted, "Never wake after a sleeping draught." At the same time there may be reasons for a most strict adherence to the directions of the bed-letter, and the night nurse should anticipate every contingency which is likely to arise, and not act upon her own responsibility in so grave a matter as omitting a medicine. As to food, the nurse may have a freer hand, and so long as the full quantity ordered is made up, it may rest with her to give it less frequently or in more concentrated form. Here she will consider the patient's good, and not spare herself some calculation and even work. So, then, night nursing may be a "speciality," but it is one which every nurse must make her own. It is a wonderful opportunity of showing disinterested love for all the suffering who need ministering to with possibly a tenderer care than is demanded by the same individuals, whilst day and the working hours make pain less near.

### The Undressing of Patients Suffering from Fractures.

### EXAMINATION QUESTIONS FOR NURSES.

### RESULT OF DECEMBER COMPETITION.

THE question was as follows:—1. How would you proceed to undress a man suffering from a simple fracture of the right forearm? 2. What difference would you make if the fracture was compound? 3. How would you proceed to undress a man with a simple fracture of the left femur? 4. What measures should you take if the fracture was compound?

### FIRST PRIZE.

*Question I.*—To undress such a patient needs very great care; he should be seated, if possible, an assistant supporting the fractured arm above and below the fracture. All unnecessary movements must be avoided, as it may produce compound fracture. The coat sleeve should be drawn gently from left side first, it will then easily slip from right side. The waistcoat, shirts, etc., being taken off in like manner, left side first. 2. If it were a compound fracture, I should cut up the seam of the coat sleeve, take sleeve right out, cut the shoulder seam, now pass the front part of coat under the right arm, then take left side off. The shoulder seam of waistcoat (right side) must be cut, and this, with the shirts taken off in similar manner. If the patient has to wait any length of time, the arm could be placed on a splint and slung to chest. A compound fracture would require careful bathing and a compress.

*Question II.*—In this case the man would be lying down. The boots and shoes should be taken off first; secondly the coat and waistcoat, beginning with right arm, the patient could raise his back just a little, the coat and waistcoat pushed under and drawn off left arm. The outer seam of left trouser leg could be cut right down; this would prevent any unnecessary movements. The trouser gently drawn from under left leg and taken off right leg in usual manner. If the fracture be compound great care must be taken. There may be an open wound into which some parts of the clothing have been forced. The thigh must be carefully sponged at seat of fracture and compress applied. A long splint placed from axilla, reaching to below the sole of the foot, and a short inner splint applied and bandaged.

REKCIV.

### SECOND PRIZE.

1. Remove the sleeve from the left arm first, then, if sufficiently loose, gently draw sleeve off the injured arm, keeping the arm in the position most comfortable to patient, taking great care not to jerk, for fear of causing the fracture to become compound. If the coat sleeve is tight, rip up outer seam; shirts and vests should be removed the same way.

2. If a compound fracture, the arm should be raised and supported; the sleeve from left arm removed first, then rip up outer seam of right sleeve and the coat will be easy to remove. The same method applies to shirt or vest sleeves.

3. Patient should be placed in the horizontal position, on a bed, if possible, coat and waistcoat removed, and all buttons undone. Boots should be removed by holding the ankles firmly, and gently drawing them off; sock should be ripped up if tight. If several to help, the trousers may be drawn off without cutting, by supporting the injured part and putting slight extension on to the leg by holding the ankle with both hands and gently pulling. If no assistance, cut the outer seam of trouser-leg and draw from under the leg towards the lined side, then the right trouser-leg can be easily removed.

4. If the fracture was compound, the seam of the trousers should always be cut, and the extent of the injury ascertained the wound should be covered up to keep aseptic, and the limb kept perfectly still by means of sand-bags Blocks may be placed under the end of the bed to raise it, if fear of hæmorrhage. When removing the clothing great care should be taken not to catch any splintered pieces of bone or disturb any clots that may have formed. MONA.

### HONOURABLE MENTION.

The three candidates who have gained honourable mention are "Experience," "Nurse Margaret," and "Nurse Ida." The paper sent by "Experience" is very good in many ways, but it is carelessly expressed and has several clerical errors. Surely it is worth while to take the trouble to read your papers through before sending them in. A little more care would probably have brought out this nurse a prize winner. Believe me, the old adage is a good one, "What is worth doing at all is worth doing well!"

### THE TWO BEST PAPERS.

"Rekciv" wins the first prize because she realises the necessary difference in treatment required in the case of a compound fracture and in that of a simple one. As she explains—by cutting up the sleeve of the injured arm and also the shoulder seam, it would be quite easy to remove the coat, however serious the injury is. With regard to a simple fracture, "Mona" is quite right in her explanation as to removing the sleeve from the sound arm *first*. Her paper is very good, but this slight inaccuracy, or, perhaps, I should say want of judgment, loses her the first prize.

### QUESTION FOR JANUARY.

What measures would you take to avoid bedsores in the case of helpless patients?

N.B.—Observe that you are not asked for treatment of the actual sores, but simply for the best method of prevention.

THE EXAMINER.

### RULES.

The competition is open to all. Answers must not exceed 500 words, and be written on one side of the paper only. The pseudonym, as well as the proper name and address, must be written on the *same* paper, and *not on a separate sheet.* Papers may be sent in for fifteen days only from the day of the publication of the question. All illustrations strictly prohibited. Failure to comply with these rules will disqualify the candidate for competition. Prizes will be awarded for the two best answers. Papers to be sent to "The Editor," with "Examination" written on the left-hand corner of the envelope.

N.B.—The decision of the examiners is final, and no correspondence on the subject can be entertained.

In addition to two prizes honourable mention cards will be awarded to those who have sent in exceptionally good papers.

### Wants and Workers.

DISTRICT NURSE, South Brent, Devon, thanks her many kind friends for offers of THE HOSPITAL.

WILL anyone having a pair of crutches to spare, kindly allow District Nurse, Cawston Lodge, Haverland Park, Norwich, to have them for a very poor woman (4 feet 3 inches in height). She has been laid aside a long time, but with the aid of crutches might regain the use of her legs. They would be gratefully received and thankfully acknowledged.

### Everybody's Opinion.

[Correspondence on all subjects is invited, but we cannot in any way be responsible for the opinions expressed by our correspondents. No communication can be entertained if the name and address of the correspondent are not given as a guarantee of good faith, but not necessarily for publication. All correspondents should write on one side of the paper only.]

### THE SCARCITY OF WORKHOUSE NURSES.

"Another Workhouse Nurse" writes: In reply to a "Trained Workhouse Matron's" criticism of my letter of December 14th on above subject, I wrote months ago to the Local Government Board and to an inspector, telling them very fully of the opposition I had from a master and matron, and asking them to consider my communication public if they wished. This was after giving up appealing to the guardians in despair. An inspector then gave me an interview, and he certainly did not consider my troubles supposed but very real. As the master and matron in question have resigned by request of those in authority, and are no longer Poor Law officials, in loyalty to the guardians and the new officials I withhold names. But what is possible to be done under present laws by a certain class of master and matron is too deeply impressed on my memory for me to forget to sympathise with nurses in the position in which I found myself, although now it is of no personal interest to me. I earnestly repeat my advice to guardians—When there is friction look at both sides fairly.

### ARMY NURSING IN SOUTH AFRICA.

"J. E. M.," of the Army Nursing Service Reserve, writes from Military Hospital, Burghersdorp, Cape Colony, under date of December 16th, 1901: On behalf of the three nursing sisters stationed at the above hospital, I am writing in reply to a letter from "An Army Reserve Sister" in The Hospital for November 16th. Since the lady in question felt "moved to write," it seems a pity she did not confine herself to a station with which she is acquainted. She is certainly not one of ourselves, nor has she ever worked in this place, and we naturally resent what she has written, as it casts a slur not only on army nursing generally, but also on the work carried on in this town, from which quarter she would give one to suppose she had gathered her altogether erroneous impressions. I came out to this country early last year, so I can speak from experience, having come across a great many nursing sisters. In such a large body of women there must be some "black sheep," but it seems to me that the majority have not only a keen sense of duty but the interests of their patients at heart. I have seen comparatively little of the "carryings on" your correspondent alludes to and equally little that might be termed "unseemly," and as in small places officers and nursing sisters are of necessity thrown so much together, it is rather hard that in the hours of relaxation intercourse cannot be indulged in without calling forth such comments as to their coming out to "amuse" the individuals in question. I am thankful I have not found the soldier patient a "severe judge" nor "over quick to think and speak evil;" quite the reverse, for I have never before met a class of men more easily pleased, more genuinely grateful, nor more loyal to those in attendance on them—in fact invariably keenly alive to the most trifling kindness and attention. With regard to the attack she makes on army nursing, I am glad to say that work is not carried out on these lines in all the hospitals out here. It is unfortunate that she has had such an experience, which, judging by her letter, must have been limited. I shall feel obliged if you will kindly put me into communication with her, and also if you will be good enough to publish my letter in order to correct those ideas which would naturally be the outcome of what she has said concerning our little hospital which we would justly defend.

[We are informed by the author of the original communication in a letter which also reaches us by the last mail, that the writer did not refer either to the sisters at Wynberg or Burghersdorp, but that her statement was made from general experience.—Ed. Hospital.]

### A PROVINCIAL HOSPITAL LEAGUE.

"The Matron of the Royal South Hants and Southampton Hospital (Miss Mollett)" writes: It may perhaps interest some of your readers to know that we have started a league of our certificated nurses on the lines of that of St. Bartholomew's Hospital. It came into existence on Friday, January 10th. The attendance at the first meeting was small, and I think that will be the great difficulty which all Provincial Hospital Leagues will experience, but the letters from those wishing to join were most enthusiastic, and we shall start with a very fair number of members. We hope to bring out our journal by Easter, and have pressed the old shield of Southampton, with its three Tudor roses, into our service for a badge. Quiet as our beginning will be, the plan evidently afforded my old nurses so much pleasure, that I hope other County Hospital matrons, not to speak of London ones, may be moved to initiate similar combinations for their old probationers.

### THE ROYAL NATIONAL PENSION FUND FOR NURSES.

"An old Mildmay Nurse, Policy 1791," writes: With your kind permission, I should like to tell the nurses that I would advise them all to join the Royal National Pension Fund for Nurses. I have been a member or policy-holder for eleven years, and during that time I have been more than satisfied. If I have wanted advice, I have gone at once to Mr. Dick, the secretary. I have often found him very busy, but never too busy to explain and make things clear to me. And now, having no need of a pension, I have withdrawn my money, which was paid to me immediately. And I am very satisfied with the interest. I am sure that nurses can find no better way of saving for old age.

### The Nurses' Bookshelf.

Surgeons and their Wonderful Discoveries: Stories of Chloroform, of the Invaluable Antiseptic Treatment, the Finsen Light, the Röntgen Rays, and other Triumphs of Surgical Skill and Practice. By F. M. Holmes, author of "Engineers and their Triumphs," "Chemists and their Wonders," etc. (London: S. W. Partridge and Co. Price 1s. 6d.)

This little book is an attempt to tell in popular form the progress of our surgery in recent times, including the work done by the latest apparatus and in the newest experiments. Strictly speaking, the last chapter should have come first, for it contains a summary of the surgical work of the past ages, with brief biographies of some eminent surgeons, such as Hippocrates, Galen, then—a great leap in point of time, but for many centuries surgery ceased to be practised on scientific lines, and fell into the hands of ignorant men, such as farriers and barbers — the great Frenchman, Ambroise Paré. Wiseman and the two Hunters are the eminent British surgeons of the past to whom most attention is given, though others receive honourable mention. But although this chapter should, as a matter of chronology, come first, it has perhaps a better chance of being read by the young people, for whom the book is chiefly intended, when it comes after their interest has been excited by the more thrilling tales of the work of Simpson, Lister, and others, whose names are familiar among us to-day. The first chapter deals with the discovery of the anæsthetic value of chloroform, from which the greater part of modern surgery dates. Some account is given of the different anæsthetics which had been already tried when chloroform came to take the place of nearly all of them, and it may be that too many chemical details are given for the taste of young folks; but this makes the little book of more substantial value to those who want an accurate, though brief, and popular account of the progress of anæsthetics. From anæsthesia to antisepsis is a natural step, and the next chapter is taken up with the discovery of the value of carbolic acid, and a narration of the method of that treatment which is specially associated with the name of Lord Lister. After this comes a description of "perfect" hospital wards, with illustrations drawn from several London hospitals. Following this come chapters dealing with those appliances by which light is made to help the surgeon's work. Almost everybody knows about the Röntgen rays, for so much has been written about them in newspapers, and examples of their power can be given in any journal; but the Finsen rays, for the treatment of lupus, are less generally known, although seeing that they were first introduced into this country by our beloved new Queen, who saw the Finsen treatment in a hospital in Copenhagen, and in consequence presented the London Hospital with a set of apparatus. It is strange that so little, comparatively, has been said about it. All this may be learned from Mr. Holmes's book, which contains a great deal of information on subjects of which but little is popularly known, but which deserves to be remembered among those achievements of science of which this generation is proud.

A SHORT MANUAL FOR MONTHLY NURSES. By CHARLES J. CULLINGWORTH, M.D., F.R.C.P. Fifth edition. Revised by the author with the assistance of MISS M. A ATKINSON, Matron of the General Lying-in Hospital. (London: J. & A. Churchill. 1901. Price 1s. 6d.)

THIS is not a guide to midwifery, but, in addition to its own proper subject, it contains enough about what happens in a confinement to enable any nurse blessed with common sense to know what to do should a child unexpectedly make its appearance before the arrival of the doctor. The intention with which the book has been written is to provide those who have already been trained in general nursing with plain directions with regard to the duties of a monthly nurse. Beginning with the early signs of pregnancy and a general outline of the process of pregnancy, it goes on to describe the management of that condition and of the various discomforts which may arise during its continuance. Then we come to a sketch of the process of natural labour and the duties of the nurse during its several stages. We are also told what to expect and what to do in the absence of the medical attendant. Then comes a chapter on the management of the child, another on the management of the mother, and finally one on antiseptics. It is a little book but it is a good one, and if read carefully will serve its purpose very well and enable a nurse to undertake the somewhat special duties of the lying-in room without nervousness.

TREATMENT OF THE INSANE, THEN AND NOW. FRIENDLY TALKS WITH A NEW PATIENT. THE NEW ASYLUM NURSE. VISITING DAY AT THE ASYLUM. AN ADDRESS TO ASYLUM ATTENDANTS. By the Rev. HENRY HAWKINS, late Chaplain of Colney Hatch Asylum. (S. P. C. K.)

THESE unpretending little books, written by one whose experience within the walls of an asylum should make him able to understand the difficulties and delicacies of the situation, are sure to be of use to those to whom they are specially addressed. They are published at 1d. each.

CASTLES AND ABBEYS OF ENGLAND AND IRELAND. Illustrated. Published Monthly. Part 2. Vol. 1. (Dicks: Strand. 6d.)

HERE we have a short history of the following places of note: Dublin Castle (continued), Tower of London, Melrose Abbey, Glastonbury, Chepstow Castle, Boyle Abbey. The letterpress and illustrations are alike excellent for the price, and the addition of legendry and romantic lore connected with the building makes an attractive relief to historical facts.

### Appointments.

BIGGLESWADE ISOLATION HOSPITAL.—Miss Fanny Dale has been appointed nurse. She was trained at Leeds Union Infirmary for three years, and has since been charge nurse at the Northern Fever Convalescent Hospital, London, charge nurse at Keighley Infirmary, and charge nurse at Kendray Hospital, Barnsley.

BOLTON INFIRMARY AND DISPENSARY.—Miss C. Griffin has been appointed sister of the male wards. She was trained at the Royal Infirmary, Derby, where she has since held the position of sister.

CONCENTRATION CAMPS, SOUTH AFRICA. — Miss A. Champley has been appointed matron of one of the concentration camps. She was trained at the Royal Infirmary, Edinburgh, where she was afterwards night nurse, and staff nurse. She has since been charge nurse, home sister, and assistant matron at the Royal Infirmary, Hull.

LAS PALMAS HOSPITAL, GRAND CANARIES.—Miss Knowles has been appointed matron. She held the position of sister at the Infirmary and Dispensary, Bolton, for several years.

NEW ISOLATION HOSPITAL, WIMBLEDON.—Miss Annie Elizabeth Reed has been appointed night sister. She was trained at Crumpsall Infirmary, Manchester, for three years, where she has since been charge nurse for nearly two years. She has also been charge nurse at the North-Eastern Fever Hospital for 18 months, charge nurse at the Park Hospital, Hither Green, for 18 months, and has done matron's holiday duty at the Blackburn Fever Hospital.

REST CONVALESCENT HOME, PORTHCAWL.—Miss Janet Lambton has been appointed matron. She was trained at the Royal Albert Edward Infirmary, Wigan, and has since been matron of Croydon Hospital, matron of Chesterfield and North Derbyshire Hospital, and matron of the Beckett Hospital, Barnsley.

ROYAL LONDON OPHTHALMIC HOSPITAL, CITY ROAD, LONDON.—Miss Maud Abraham has been appointed night sister. She was trained at the Cardiff Infirmary for three years, and has since held the posts of staff nurse at the Royal London Ophthalmic Hospital, charge nurse at the Hertford British Hospital, Paris; and charge nurse at the Western Fever Hospital, Fulham.

STROOD UNION INFIRMARY.—Miss Kathleen Josephine Bowen has been appointed night nurse. She was trained for maternity only at St. Francis Maternity and Nursing Home, Commercial Road, Peckham, and has since been head nurse and midwife at Dorking Union, Norfolk. She holds the L.O.S. certificate.

### Presentations.

KENT NURSING INSTITUTION, BROMLEY.—The nurses of the Kent Nursing Institution, Bromley, which is to be closed on the 8th of next month, have presented Miss A. Hulton-Potts with a complete solid silver toilet set, on her birthday, January 4th, as a token of their appreciation of the kindness which she has shown to them during the three years she has held the post of lady superintendent.

WOODFORD JUBILEE HOSPITAL, WOODFORD GREEN.—On Christmas Eve Miss Amy Yarrow, matron of Woodford Jubilee Hospital, was presented with a beautiful etching—"Against Wind and Open Sky"—by the members of the ladies' committee. This was particularly gratifying to the recipient, as she has held the post for scarcely a year.

### Novelties for Nurses.

LAST week we noticed Viyella specialities under the title of blankets. We should have described them as woollen sheets, as they are intended to be used as such.

### For Reading to the Sick.

EPIPHANYTIDE.

"*When they saw the star they rejoiced with exceeding great joy.*"

As with gladness men of old
Did the guiding star behold,
As with joy they hailed its light
Leading onward, beaming bright;
So, most gracious Lord, may we
Evermore be led to Thee.

Holy Jesus every day
Keep us in the narrow way,
And when earthly things are past
Bring our ransomed souls at last
Where they need no star to guide
Where no clouds thy glory hide.

*C. Dix.*

The manifestation of Christ to the soul leads to the recognition of a vocation in life. The Magi saw the Star, and it called them to leave their country, to undertake a long journey to seek Christ in Bethlehem. Like St. Paul, when we have heard the voice of Jesus, we must ask, "Lord, what wilt Thou have me to do?"—*A. G. Mortimer.*

This sacred Name—Jesus, has power over all nature. It has restored the dead to life, cured all manner of sickness and disease, and filled the world with miracles.

Should I be afflicted in body myself, or should I be distrest for those in sickness, I will call upon the Name of the Lord Jesus. I will plead with God by that Name: I will offer it to Him in prayer and in the Holy Eucharist: and I will believe that nothing is impossible which is asked in the Name of Jesus.—*Anon.*

It is the time of darkness that Christ has ever lifted up His light. Yea, as night itself brings the stars to sight, so in our darkness of adversity come forth more clearly to view the eternal things of Heaven. Whence it hath passed, as it were, into a sacred proverb of all affliction, "When I sit in darkness, the Lord shall be a light unto me." Micah vii. 8.—*Rev. Isaac Williams.*

All the souls, everywhere, in whom God dwells, dwell together in virtue of that indwelling. They may be separated very far. They may not know each other's tongue. The Divine presence in them may take the most utterly various forms of expression. Their works in life may be entirely distinct. All these are things external. They live together as they both abide in God. The symbols of that inner life are many; the multitudinous life itself is one. . . . The Communion of Saints is a mutual ministry of saints.

*Phillips Brooks, Sermons.*

Brightest star of eastern skies,
Let that final morn appear,
When our bodies too shall rise,
Free from all that pain'd them here,
Strong their joyful course to run
As the sun.

To yon world be Thou our light,
O Thou glorious Sun of grace;
Lead us through the tearful night,
To yon fair and blessed place;
Where to joy that never dies
We shall rise.

*Von Rosenroth.* 1684.

### Notes and Queries.

**The Editor is always willing to answer in this column, without any fee, all reasonable questions, as soon as possible.**

**But the following rules must be carefully observed:—**

**1. Every communication must be accompanied by the name and address of the writer.**

**2. The question must always bear upon nursing, directly or indirectly.**

**If an answer is required by letter a fee of half-a-crown must be enclosed with the note containing the inquiry.**

*Hospital* v. *Nurses' Homes.*

(149) After working for an institution I want to return to a hospital affiliated with the R.N.P.F.N. Can you give me a list? I very much prefer working for a hospital at £30 a year than for an institution for which I make £2 10s. weekly and receive for myself 12s. 6d. In a hospital a nurse has her day in the week and her month's holiday in the year, and she is sure of her night's rest after her day's work is done.—*M. C. F.*

Write to the Secretary of the Royal National Pension Fund for Nurses, 28 Finsbury Pavement, E.C. for a list.

*Cottage Nurse.*

(150) A strong girl, 25, with great aptitude for the care of the sick, is desirous of becoming a cottage nurse. As she has only a very elementary education, it is difficult to get her into a training institution. Can you tell me where I can find out about the scheme for supplying cottage nurses?—*M. E. A.*

Apply to the Secretary, the Affiliated Benefit Nursing Associations, 12 Buckingham Palace Road, S.W.

*Cap.*

(151) Is it usual for a nurse to wear her cap whilst on a visit to her friends?—*D. H.*

No. Uniform is generally only worn when nursing.

*Break-down.*

(152) If a nurse, having signed for a three years' training at a large London hospital, breaks down at the end of the second year; and if the medical man gives it as his opinion that she will be fit to resume work after a year's rest, can the matron prevent her from returning at the end of that time, if she herself be willing to take the responsibility?—*M. S.*

The matron is generally supreme in matters of this kind. The best thing, under the circumstances, would be for the nurse to call upon her matron when her strength is fully re-established, and try and obtain the matron's sanction to the completion of her training.

*Homes.*

(153) Can you tell me of a home for an imbecile youth of eighteen? He cannot walk, but has the use of his hands and eyes, and might be taught a trade such as basket making.—*Rev. W. J.*

Apply to the Secretary the National Association for Promoting the Welfare of the Feeble-minded, 53 Victoria Street, S.W.

Could you kindly tell me of a home where a poor woman suffering from nervous depression after influenza could be admitted either permanently or for a time. She could pay from three to five shillings weekly.—*A. M.* (Cornwall).

The Royal West of England Sanatorium, Weston-super-Mare, would receive the case at that fee if you could get a letter.

Will you kindly tell me of a home where a girl subject to epileptic fits could be received free of charge? It is a case of extreme poverty.—*Nurse L. G.*

Is not this a case for which the guardians should be responsible? See reply to *Rev. W. J.*

*Accouchement.*

(154) Will you kindly tell me of any apartments especially intended for accouchement cases? The lady who requires them would take her own nurse, so that only rooms and attendance would be needed.—*H. M.*

We do not recommend private homes, but doubtless there are many which would be quite willing to make the required arrangement, and we should advise you to advertise.

**Standard Books of Reference.**

"The Nursing Profession: How and Where to Train." 2s. net; post free 2s. 4d.
"Burdett's Official Nursing Directory." 3s. net; post free, 3s. 4d.
"Burdett's Hospitals and Charities." 5s.
"The Nurses' Dictionary of Medical Terms." 2s.
"Burdett's Series of Nursing Text-Books." 1s. each.
"A Handbook for Nurses." (Illustrated). 5s.
"Nursing: Its Theory and Practice." New Edition. 3s. 6d.
"Helps in Sickness and to Health." Fifteenth Thousand. 5s.
"The Physiological Feeding of Infants." 1s.
"The Physiological Nursery Chart." 1s.; post free, 1s. 3d.
"Hospital Expenditure: The Commissariat." 2s. 6d.

All these are published by the SCIENTIFIC PRESS, Ltd., and may be obtained through any bookseller or direct from the publishers, 28 and 29 Southampton Street, London, W.C.

## Figures and Tables

**Fig. 20. f1:**